# Solving Integer Ambiguity Based on an Improved Ant Lion Algorithm

**DOI:** 10.3390/s25041212

**Published:** 2025-02-17

**Authors:** Wuzheng Guo, Yuanfa Ji, Xiyan Sun, Xizi Jia

**Affiliations:** 1 Information and Communicaiton School, Guilin University of Electronic Technology, Guilin 541004, China; gwz2095949183@163.com (W.G.); sunxiyan1@163.com (X.S.); 18907830034@163.com (X.J.); 2 Guangxi Key Laboratory of Precision Navigation Technology and Application, Guilin University of Electronic Technology, Guilin 541004, China; 3 International Joint Research Laboratory of Spatio-Temporal Information and Intelligent Location Services, Guilin University of Electronic Technology, Guilin 541004, China; 4 GUET-Nanning E-Tech Research Institute Co., Ltd., Nanning 530031, China

**Keywords:** global navigation and positioning system, double-difference carrier phase, integer ambiguity, higher-dimensional ambiguity resolution, centimeter-level accuracy positioning

## Abstract

In GNSS, a double-difference carrier phase observation model is typically employed, and high-accuracy position coordinates can be obtained by resolving the integer ambiguity within the model through algorithmic processing. To address the challenge of a double-difference integer ambiguity resolution, an enhanced Simulated Annealing Ant Lion Optimizer (SAALO) is proposed. This algorithm is designed to efficiently resolve integer ambiguities. First, the performance of the SAALO algorithm was evaluated by comparing its solving speed and success rate with those of the Ant Lion Optimization Algorithm (ALO), the LAMBDA algorithm and the MLAMBDA algorithm. The results demonstrate that the SAALO algorithm achieved a solution success rate that was 0.0496 s and 0.01 s faster than the LAMBDA and M-LAMBDA algorithms, respectively. Second, to further validate the high-dimensional ambiguity resolution capability of the SAALO algorithm, integer ambiguity resolution tests were conducted in both 6-dimensional and 12-dimensional scenarios. The results indicate that the SAALO algorithm achieves a success rate exceeding 98%, confirming its robust performance in high-dimensional problem-solving. Finally, the practical application of the SAALO algorithm was tested in short- and medium-baseline scenarios using a single-frequency GPS system. With a baseline length of 42.7 km, the SAALO algorithm exhibited a slightly faster average solution time compared to the LAMBDA algorithm, while its solution success rate was 5.2% higher. These findings underscore the effectiveness and reliability of the SAALO algorithm in real-world GNSS applications.

## 1. Introduction

In the Global Positioning System (GPS) positioning mode, the ability to achieve centimeter-level or even millimeter-level positioning accuracy using carrier phase observations hinges on the correct and efficient resolution of integer ambiguity [1]. Integer ambiguity resolution is a fundamental challenge in carrier phase differential measurements and serves as the core problem that must be addressed to attain high-precision positioning results.

Since the integer ambiguity resolution algorithm was proposed in the 1980s; it has been studied by many scholars at home and abroad. Genetic Algorithm (GA) is a method to find the optimal solution by simulating the natural evolutionary process and obtaining the optimal solution through selection, crossover, mutation and other operations. When applied to the problem of searching for the optimal integer ambiguity resolution, Genetic Algorithm features global optimization, robustness, and high efficiency. However, simultaneously, it also confronts issues like a larger search space, more complex computations and a tendency to easily fall into local optima [2]. The LAMBDA (Least-Square Ambiguity Decorrelation Adjustment) algorithm utilizes the integer ambiguity floating-point resolution and its covariance to determine the optimal integer ambiguity resolution. It constructs the cost function and the ambiguity search space based on the ambiguity floating-point resolution and its covariance matrix, and then conducts a traversal search within the search space to solve for the integer ambiguity [3,4]. The ambiguity resolution (AR) model mainly consists of two parts: integer estimation and validation. The main integer estimation methods include integer bootstrap, integer rounding, and Integer Least Squares (ILS) [5]. With the development of the ambiguity estimation theory, Teunissen introduced the Integer Aperture (IA) estimator for carrier phase AR [6]. Teunissen derived the Optimal Integer Aperture (OIA) estimator, which achieves the highest success rate for a user-defined failure rate within class IA. Although OIA has the highest theoretical success rate, its practical application encounters challenges because a large amount of sample statistics are needed for threshold computation [7]. Wu and Bian developed a validation method based on posteriori probability, which is equivalent to OIA in terms of expression. Additionally, incorporating supplementary constraint information into the GNSS measurement model can also facilitate integer ambiguity verification [8]. There is an enhanced integer least-squares estimation method: the MLAMBDA (Modified Least-square Ambiguity Decorrelation Adjustment). The computational efficiency of the search phase is improved by reducing the complexity of the method in the search phase [9]. An improved flock optimization algorithm based on the Improved Chicken Swarm Optimization (ICSO) algorithm was used to solve integer ambiguity problems, and this enhanced flock optimization algorithm was applied to the search for integer ambiguities [10]. The ambiguity search was carried out using an improved Particle Swarm Optimization (PSO) algorithm [11]. An improved hybrid algorithm for GNSS integer ambiguity search that combines the particle swarm algorithm and the Ant Colony Optimization (ACO) algorithm is proposed. In the initial stage of the search, an enhanced particle swarm optimization algorithm was used to obtain a suboptimal solution, which served as an initialization of the pheromone distribution of the improved ACO algorithm. Eventually, an accurate search for integer ambiguity was achieved [12]. The effectiveness of the ACO algorithm in solving ambiguity and nearest grid point problems was investigated by quickly solving the GNSS ambiguity solving problem through ACO optimization [13]. An enhanced artificial swarm technique with additional integer constraints was studied to find fast stationary solutions [14]. An enhanced LAMBDA method is proposed to improve the efficiency of ambiguity solving. It modified the previous method of searching all the ambiguities for each epoch. This study effectively combined search and normalization under reasonable conditions to enhance the determination of ambiguity after z-transformation. These studies improved the efficiency of the algorithm by optimizing the search conditions, the LAMBDA algorithm and the search space. The algorithm was theoretically rigorous. Although it had a high fixation success rate, it was computationally very long and its search efficiency decreased as the ambiguity dimension increased [15]. The basic idea was to compute the corresponding objective function value as soon as a new population of ambiguity vectors is obtained during the search process. If the newly computed value is smaller than the previous one, the new value will replace the previous objective function value. In this way, the search space is gradually reduced and the optimal streaming vector population is finally obtained [16]. A DGPS (Differential Global Positioning System) integer ambiguity resolution algorithm based on an improved particle swarm optimization algorithm is proposed. This algorithm uses the inertia degradation method of the sine function to adjust the weights of the particle swarm optimization [17]. When the floating-point solution and its variance covariance matrix are not precise enough, constraints based on known conditions are introduced to obtain accurate attitude information. Extending the search ellipsoid region to compensate for errors caused by inaccuracies in the floating-point solution [18], a method based on the simulated annealing genetic algorithm is proposed to solve the integer ambiguity problem. This method uses an improved genetic algorithm to search for ambiguities in the entire search space and finally obtains the optimal solution [19]. AWDE (Adaptive Weighting Differential Evolution Algorithm) is proposed to adaptively adjust the difference operator in the DE algorithm, aiming to avoid getting trapped in local optima and improve the solution efficiency [20]. In integer ambiguity search, the construction of the cost function and the nature of the search space can be viewed as a nonconvex optimization problem in the mathematical field [21], a heuristic optimization algorithm can be used to fix the integer ambiguities.

To address the issues of a wide search range, low search efficiency and a tendency to get trapped in local optima when resolving integer ambiguity, an improved Ant Lion Optimizer (ALO) algorithm is proposed for integer ambiguity resolution. This algorithm shortens the ambiguity search time, prevents the algorithm from falling into local optima and enhances the solution success rate.

## 2. GNSS Differential Positioning Principle

### 2.1. Double-Difference Observation Equations

In RTK (Real-Time Kinematic) positioning technology, at least two receivers are required to simultaneously observe signals from multiple satellites [22]. Assuming that the reference station receiver is defined as b and the mobile station receiver is defined as r, and that the two receivers receive signals from satellite i and satellite j simultaneously, the carrier phase double-difference observation equation can be established as follows:

(1) $$\lambda \phi_{br}^{i,j}=\rho_{br}^{i,j}-i_{br}^{i,j}+t_{br}^{i,j}+\lambda b_{br}^{i}-\lambda b_{br}^{j}+\varepsilon_{br}^{i,j}$$

In Equation (1), λ is the carrier wavelength value; $\phi_{br}^{i,j}$ is the carrier phase double difference between the two receivers and the observed satellites; $\rho_{br}^{i,j}$ is the actual distance double difference between the two receivers and the observed satellites; $i_{br}^{i,j}$ is the ionospheric delay double difference; $t_{br}^{i,j}$ is the tropospheric delay double difference; $b_{br}^{i},b_{br}^{j}$ is the single-difference value of integer ambiguity; and $\varepsilon_{br}^{i,j}$ is the combined value of other errors.

After the carrier phase observations are double-difference calculated, the common errors in the troposphere and ionosphere observations were basically eliminated, and the residual values can be summarized in other error terms: $\varepsilon_{br}^{i,j}$. Therefore, the carrier phase observations can be combined into other error terms, and the residual values can be summarized into other errors. Therefore, the carrier phase observations can be combined with other error terms, and residual values can be grouped into other errors. Consequently, Equation (1) can be abbreviated as follows:

(2) $$\lambda \phi_{br}^{i,j}=\rho_{br}^{i,j}+\lambda n_{br}^{i,j}+\varepsilon_{br}^{i,j}$$

In Equation (2), $n_{br}^{i,j}$ is the Double-Difference Integer Ambiguity.

### 2.2. Cost Loss Function

In the RTK positioning process, Least Squares (LS) or Extended Kalman Filter (EKF) is usually used to solve the double-difference observation equation. The standard least-squares solution mainly takes into account the data obtained at the current time. This approach separates the time correlation in satellite positioning and is susceptible to noise and other errors. The extended Kalman filter not only considers the data information at the current moment, but also integrates the positioning results of the previous moment, which can more accurately reflect the position of the object [23]. The EKF has more advantages in solving the double-difference observation equations for the Double-Difference Integer Ambiguity Floating-Point Values (DIAFV) and their covariance matrices.

In EKF, Equation (1) is linearized to yield the following state equation:

(3) y=Bb+An+ε

In Equation (3), y is the vector of carrier phase double-difference observations; b is the baseline vector between the two receivers; B is the corresponding coefficient matrix; n is the vector of ambiguity parameters to be solved; A is the corresponding coefficient matrix; and ε is the vector of residuals for the observations.

The noise ε is assumed to be Gaussian zero-mean with a covariance [24]

(4) $$Q_{y}=E\left[\varepsilon \varepsilon^{T}\right]$$

In the case of undifferenced data, the matrix is generally diagonal. It is assumed that the variance of phase measurements remains uniform across all carriers, while the code measurement components vary due to the differing code rates of the signals. The introduction of differenced data creates a strong correlation in the statistical properties.

The primary objective of the navigation is to estimate the baseline vector b and resolve the integer ambiguities n. Under Gaussian assumptions and in the absence of prior information, this can be formulated as a least-squares optimization problem:

(5) $$\min{\left\|y-An-Bb\right\|}_{{Q^{-1}}_{y}}^{2},n\in Z^{k},b\in R^{k}$$

The latter norm can be split:

(6) $$\begin{array}{ll} {\left\|y-An-Bb\right\|}_{{Q^{-1}}_{y}}^{2} & ={\left\|P_{B}\left(y-An\right)-Bb\right\|}_{{Q^{-1}}_{y}}^{2}\\ & \;+{\left\|\overset{⌢}{n}-n\right\|}_{{Q^{-1}}_{\overset{⌢}{n}}}^{2}+{\left\|P\frac{1}{\bar{A}}P\frac{1}{B}\right\|}_{{Q^{-1}}_{y}}^{2} \end{array}$$

By introducing the projectors

(7) $$\begin{array}{l} P_{B}=B{\left(B^{T}Q_{y}^{-1}B\right)}^{-1}B^{T}Q_{y}^{-1},\\ P_{\bar{A}}=\bar{A}{\left({\bar{A}}^{T}Q_{y}^{-1}\bar{A}\right)}^{-1}{\bar{A}}^{T}Q_{y}^{-1} \end{array}$$

the float ambiguity

(8) $$\overset{⌢}{n}={\left({\bar{A}}^{T}Q_{y}^{-1}\bar{A}\right)}^{-1}{\bar{A}}^{T}Q_{y}^{-1}y$$

And the correlated noise metric for n

(9) $$Q_{\overset{⌢}{n}}^{-1}={\bar{A}}^{T}Q_{y}^{-1}\bar{A}$$

The projection on the image of B is the first term of (6). The last term neither depends on b nor n, and is thus an irreducible error.

The core task is to find

(10) $$\begin{array}{ll} J\left(n\right) & =\arg\min{\left\|\overset{⌢}{n}-n\right\|}_{Q_{\overset{⌢}{n}}^{-1}}^{2}\\ & =\min[{\left(n-\overset{⌢}{n}\right)}^{T}Q_{\overset{⌢}{n}}^{-1}\left(n-\overset{⌢}{n}\right)] \end{array}$$

Since the ambiguities must be integers, the result will not be truly infinite large. The equipotential surfaces of the associated metric are extremely elongated ellipsoids. If the ambiguity search is conducted directly without the decorrelation operation, it will lead to an excessively long search time due to the large amount of computation.

In satellite navigation, the carrier waves received by the antennas at both ends of the baseline are coherent waves with the same frequency, vibration direction, and a fixed-phase difference [25].

**Figure 1:**
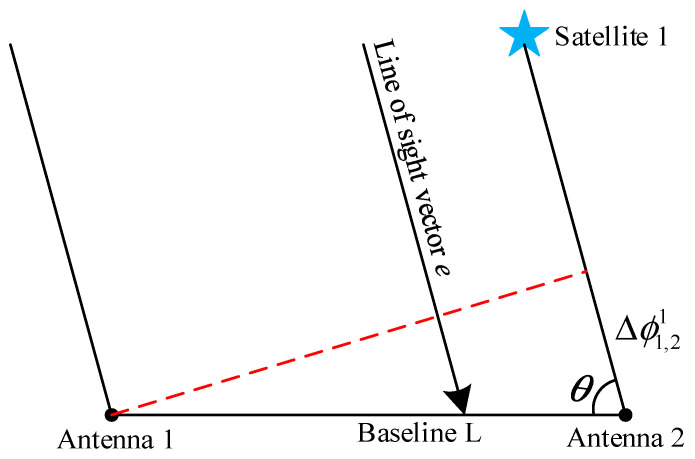
Schematic diagram of the antenna receiving carrier.

As presented in Figure 1 below, θ is the angle between the unit line-of-sight vector e and the baseline vector $l_{m}$. The following single-differential carrier phase equation can be established:

(11) $$\Delta \phi_{1,2}^{1}=\lambda^{-1}l_{m}\cos\theta +n_{1,2}^{1}$$

In Equation (11), $\Delta \phi_{1,2}^{1}$ is the single-difference carrier phase and $n_{1,2}^{1}$ is the single-difference integer ambiguity.

The following double-difference carrier phase equation can be obtained:

(12) $$\Delta \phi_{1,2}^{i,j}=\lambda^{-1}l_{m}(\cos\theta_{i}-\cos\theta_{j})+n_{1,2}^{i,j}$$

According to the GNSS coherent posing principle [26], $\left|\lambda \cdot \Delta n_{1,2}^{i,j}\right|$ should be less than $\left|l_{m}\right|$, and substituting this relation into Equation (12), the baseline constraint relation equation for the double-difference integer ambiguity can be obtained:

(13) $$-l_{m}/\lambda \leq n_{1,2}^{i,j}\leq l_{m}/\lambda$$

The essence of optimization in integer ambiguity resolution is to find a set of solution vectors within the search space that can minimize the objective function. Therefore, the search space must encompass the correct integer ambiguity resolutions. The search space can be constructed based on the baseline length [27]. It is determined from the baseline constraint relation for the double-difference integer ambiguity in Equation (14) as follows:

(14) $$[{\overset{⌢}{n}}_{i}]-l_{m}/\lambda \leq n_{i}\leq [{\overset{⌢}{n}}_{i}]+l_{m}/\lambda ,i=1,2\cdots k$$

In Equation (14), $\left[{\overset{⌢}{n}}_{i}\right]$ is the integer value of the floating-point solution $\overset{⌢}{n}$ taken in the ith dimension, and $l_{m}/\lambda$ is the amplitude of the search space determined through the baseline length, using the GPS interferometry principle, e.g., a 2.0 m long baseline l, with an L1 carrier wavelength of 19.03 cm, which can be computed to be $\left[l/\lambda \right]=6$, where l is the baseline length and $\left[l/\lambda \right]$ denotes upward rounding. Therefore, the boundary condition is determined as $x_{\max,j}=[z_{j}]+6,x_{\min,j}=[z]-6$. If the baseline length is measured with a large error, it will result in a larger search space and redundant candidate values. The SAALO algorithm proposed in the following section features a high search efficiency and good accuracy. It can still yield a satisfactory solution even when the search space is large.

### 2.3. Ambiguity Decorrelation

When there is a strong correlation among the ambiguity parameters, making small adjustments to one ambiguity will cause the remaining ambiguities to interact with each other, making it difficult to converge quickly. However, if we can find ways to reduce the correlation among ambiguity parameters and minimize the impact of changes in one ambiguity on other ambiguity values, we can significantly accelerate the ambiguity search process.

Therefore, to enhance the search efficiency, the method employed in this article is the Z-transform method mentioned in references [28,29].

The core idea is to improve the search efficiency by employing the integer transformation matrix Z to perform the descending correlation of $Q_{\overset{⌢}{n}}$, thus effectively reducing the number of nodes outside the search ellipsoid. It is assumed that $Q_{\overset{⌢}{n}}$ is integer transformed and decomposed according to L’DL as shown in Equation (15).

(15) $$Q_{\overset{⌢}{Z}}=Z^{T}Q_{\overset{⌢}{n}}Z={\bar{L}}^{T}\bar{D}\bar{L}$$

where $Q_{\overset{⌢}{Z}}$ is the integer-transformed variance covariance matrix; $\bar{L}$ is the unit lower triangular matrix and the lower triangular element is ${\bar{l}}_{i,j},i>j$; $\bar{D}$ is the diagonal matrix and the diagonal element is ${\bar{d}}_{i}$, i∈[1,n], which is often called the “conditional variance”.

To improve the search efficiency requires that the ambiguity degree variance–covariance array $Q_{\overset{⌢}{n}}$ satisfy the following two constraints:

(1) $Q_{\overset{⌢}{n}}$ is diagonalized whenever possible;

(2) Conditional variances are sorted in ascending order whenever feasible.

To meet the above two constraints, integer Gaussian elimination and the exchange of neighboring conditional variances are, respectively, employed to implement them.

First, Gaussian elimination is performed on the nontriangular elements in L. Assume that any lower triangular element is $l_{i,j}(i>j)$. When its absolute value is greater than 0.5, the corresponding integer transformation matrix is $Z_{i,j}$, as shown in Equation (16).

(16) $$Z_{i,j}=I_{n}-{\left[l_{i,j}\right]}_{round}e_{i}e_{j}^{T}$$

where $I_{n}$ is the n-dimensional unit square matrix; ${\left[\right]}_{round}$ is the rounding symbol; and $e_{i},e_{j}$ is the n-dimensional unit coordinate vector.

The integer transformation of L, i.e., $\bar{L}=Z_{i,j}L$, is performed, at which time the lower triangular matrix elements need to be updated, as shown in Equation (17).

(17) $${\bar{l}}_{i,k}=l_{i,k}-{\left[l_{i,j}\right]}_{round}l_{i,k},i>j>k$$

Next, the neighboring conditional variances in D are ranked. Assume that $Q_{\overset{⌢}{n}}$ undergoes $LDL^{T}$ decomposition as shown in Equation (18):

(18) $$Q_{\overset{⌢}{n}}=LDL^{T}=\left[\begin{array}{ccc} L_{11} & & \\ L_{21} & L_{22} & \\ L_{31} & L_{32} & L_{33} \end{array}\right]\left[\begin{array}{ccc} D_{11} & & \\ & D_{22} & \\ & & D_{33} \end{array}\right]\left[\begin{array}{ccc} {L^{T}}_{11} & {L^{T}}_{21} & {L^{T}}_{31}\\ & {L^{T}}_{22} & {L^{T}}_{23}\\ & & {L^{T}}_{33} \end{array}\right]$$

When $d_{i}+l_{i,i-1}^{2}d_{i-1}<d_{i-1}$ is satisfied, the neighboring conditional variances $\left(d_{i-1},d_{i}\right)$ are exchanged. Assume that the exchange matrix is $P_{i-1,i}$, as shown in Equation (19):

(19) $$P_{i-1,i}=\left[\begin{array}{ccc} I_{i-2} & & \\ & P & \\ & & I_{n-i} \end{array}\right]$$

where $P=\left[\begin{array}{cc} 0 & 1\\ 1 & 0 \end{array}\right]$; $I_{i-2}$ and $I_{n-i}$ are unit matrices, respectively.

The integer transformation of $LDL^{T}$ using $P_{i-1,i}$ to $Q_{\overset{⌢}{n}}$ and decomposition according to A is shown in Equation (20):

(20) $$\begin{array}{ll} P_{i-1,i}Q_{\overset{⌢}{n}}{P_{i-1,i}}^{T} & =P_{i-1,i}LDL^{T}{P_{i-1,i}}^{T}\\ & =\left[\begin{array}{ccc} L_{11} & & \\ {\bar{L}}_{21} & {\bar{L}}_{22} & \\ L_{31} & {\bar{L}}_{32} & L_{33} \end{array}\right]\left[\begin{array}{ccc} D_{11} & & \\ & {\bar{D}}_{22} & \\ & & D_{33} \end{array}\right]\left[\begin{array}{ccc} {L^{T}}_{11} & {{\bar{L}}^{T}}_{21} & {L^{T}}_{31}\\ & {{\bar{L}}^{T}}_{22} & {{\bar{L}}^{T}}_{23}\\ & & {L^{T}}_{33} \end{array}\right] \end{array}$$

In Equation (20),

(21) $$\left\{\begin{array}{c} {\bar{L}}_{21}=PL_{21}\\ {\bar{L}}_{32}=L_{32}A \end{array}\right.,{\bar{L}}_{22}=PL_{22}A=\left[\begin{array}{cc} 1 & \\ {\bar{l}}_{i,i-1} & 1 \end{array}\right],D_{22}=\left[\begin{array}{cc} {\bar{d}}_{i-1} & \\ & {\bar{d}}_{i} \end{array}\right]$$

Among them,

(22) $${\bar{d}}_{i-1}=d_{i}+{l^{2}}_{i,i-1}d_{i-1};{\bar{d}}_{i}=\frac{d_{i}d_{i-1}}{d_{i-1}};A=\left[\begin{array}{cc} {\bar{l}}_{i,i-1} & 1\\ \frac{d_{i}}{d_{i-1}} & -l_{i,i-1} \end{array}\right];{\bar{l}}_{i,i-1}=\frac{l_{i,i-1}d_{i-1}}{d_{i-1}}$$

Through the aforementioned transformation process, the mathematical expression that meets the above two constraints is ultimately obtained, as shown in Equation (23).

(23) $$\left\{\begin{array}{l} \left|l_{i,j}\right|\leq 0.5,i>j\\ d_{i}+{l^{2}}_{i,i-1}d_{i-1}\geq d_{i-1} \end{array}\right.$$

In Equation (23), the first condition is referred to as elemental descending correlation, while the second is the ordering of conditional variance.

Similarly, by applying the aforementioned descending correlation method to reduce the degree of correlation among the ambiguity parameters, we can minimize the impact of the change in one ambiguity on the values of other ambiguities. This can significantly accelerate the ambiguity search process, as shown in Equation (24).

(24) $$\left\{\begin{array}{c} n_{Z}=Z^{T}n\\ {\overset{⌢}{n}}_{Z}=Z^{T}\overset{⌢}{n}\\ Q_{\overset{⌢}{Z}}=Z^{T}Q_{\overset{⌢}{n}}Z \end{array}\right.$$

In Equation (24), Z is the transformation matrix, $n_{Z}$ and $Q_{\overset{⌢}{Z}}$ are the vectors of n and $Q_{\overset{⌢}{n}}$ after integer transformation, respectively, and $n_{Z}$ is the floating-point solution of $\overset{⌢}{n}$ after integer transformation. Equation (10) is converted to a new least-squares problem:

(25) $$J\left(n_{Z}\right)=\min[{\left(n_{Z}-{\overset{⌢}{n}}_{Z}\right)}^{T}Q_{\overset{⌢}{Z}}^{-1}\left(n_{Z}-{\overset{⌢}{n}}_{Z}\right)]$$

In Equation (25), $n_{Z}$ is the integer resolution of double-difference integer perimeter ambiguity after fixation; ${\overset{⌢}{n}}_{Z}$ is the floating-point resolution of double-difference integer perimeter ambiguity after Z-transformation; $Q_{\overset{⌢}{Z}}^{-1}$ is the inverse matrix of the corresponding covariance matrix after Z-transformation.

Reducing the correlation among the covariance matrices through the Z-transformation can shorten the ambiguity search time. However, the Z-transformation itself increases the overall computational time for ambiguity resolution. Therefore, to further enhance the search efficiency, it is necessary to employ an efficient search algorithm to achieve the rapid fixation of the ambiguities.

## 3. Simulated Annealing Ant Lion Optimization Algorithm Ambiguity Search

### 3.1. Standard Ant Lion Optimization Algorithm

The Ant Lion Optimizer (ALO) was proposed by Mirjalili in 2015. It primarily emulates the predation strategy of antlions hunting ants to identify the optimal solution [30].

Step 1: Ants choose a target ant lion trap. Upon initialization, n ant lion as well as n ants are randomly initialized in the solution space with position $x=(x^{1},x^{2},\cdots ,x^{D})$. Each ant chooses a target ant lion for subsequent random wandering.

Step 2: Calculate the size of the trap. This size will be used in the next step to determine the position of the ants after their random wandering. The formula for the trap size is as follows:

(26) $$ratio=\left\{\begin{array}{l} 1,t<0.1t_{max}\\ 1+100\times \frac{t}{t_{max}},0.1t_{max}\leq t<0.5t_{max}\\ 1+1000\times \frac{t}{t_{max}},0.5t_{max}\leq t<0.75t_{max}\\ 1+10000\times \frac{t}{t_{max}},0.75t_{max}\leq t<0.9t_{max}\\ 1+100000\times \frac{t}{t_{max}},0.9t_{max}\leq t<0.95t_{max}\\ 1+1000000\times \frac{t}{t_{max}},0.95t_{max}\leq t<t_{max} \end{array}\right.$$

In Equation (26), t is the current number of iterations and $t_{max}$ is the maximum number of iterations.

Step 3: The ant wanders randomly and defines the function as rw:

(27) $$rw=\left\{\begin{array}{c} -1,r\leq 0.5\\ 1,r>0.5 \end{array}\right.$$

In Equation (27), r is a uniform random number within (0,1). The random wandering function is

(28) $$rw(t)=\sum_{i=0}^{t}rw$$

Step 4: Calculate the relative value of random wandering of ants, the value of random wandering of the ith ant at generation t is $rw_{i(t)}$.

Then, $rw_{i(t+1)}=rw_{i(t)}+rw$, the maximum value of random wandering in the population of generation t is rw(t)_max, and the minimum value is rw(t)_min. Define $arw_{i(t)}$ to be the relative position of random wandering of the ith ant at generation t. The calculation formula is

(29) $$arw_{i}^{t}=\frac{rw_{i}^{t}-(rw^{t})\_min+1}{(rw^{t})\_max-(rw^{t})\_min+1}$$

Step 5: Calculate the ant lion trap range. The ant chooses k ant lions as its target ant lions, and from the ratio calculated in the previous step, the trap range trap where the ant lions are located can be calculated:

(30) $$\begin{array}{l} trap_{k}^{d}\_min=\frac{d_{min}}{ratio}+x\_al_{k}^{d}\\ trap_{k}^{d}\_max=\frac{d_{max}}{ratio}+x\_al_{k}^{d}\\ trap_{k}^{d}\in [trap_{k}^{d}\_min,trap_{k}^{d}\_max] \end{array}$$

In Equation (30), $d_{min}$ is a lower bound for the dth dimensional solution space and $d_{max}$ is the upper bound of the range of values of the dth dimensional solution space. $x\_al_{k}^{d}$ is the dth dimensional position of the kth ant lion. $Trap_{k}^{d}$ denotes the dth dimensional range of values of the kth ant lion trap.

Step 6: Calculate the positions of the ants. The ants will wander randomly around the ant lion they choose and eventually stay within the trap of the selected ant lion. The formula for calculating the positions of the ants is as follows:

(31) $$\begin{array}{ll} x\_a_{i,k}^{d}= & arw_{i}^{t}\times (trap_{k}^{d}\_max-trap_{k}^{d}\_min)\\ & +trap_{k}^{d}\_min \end{array}$$

In Equation (31), the position of the ith ant is in the ant lion trap it chooses, and the exact position is determined by the relative position of the ant in the colony as a result of the ant’s random wandering. In addition, the ant will also wander randomly toward the globally optimal ant lion individual and eventually stay at the mid-point between the two positions:

(32) $$x\_a_{i}^{d}=\frac{x\_a_{i,k}^{d}+x\_a_{i,best}^{d}}{2}$$

Step 7: The ant lion moves to the position of the designated ant. The ant lion preys on the ant, retains the current optimal value and rebuilds the trap to continue foraging in search of a better value.

### 3.2. Simulated Annealing Ant Lion Optimization Algorithm

In the standard ant lion optimization algorithm, ants wander randomly, representing the trial solutions. The traps move toward the ant lions, which represent the local optimal solutions. After each update of all the ant lions, one of the optimal ant lions is selected as the elite ant lion, representing the global optimal solution. This algorithm has a certain global search ability, but it requires numerous iterative calculations and has a limited scope of applicability. Moreover, the standard ant lion optimization algorithm converges too rapidly. This high convergence speed may cause the algorithm to fall into local optima prematurely in some cases, posing a great challenge to the algorithm’s stability and its ability to search for the optimum.

In this paper, taking into account the actual situation in the process of integer ambiguity resolution, the SAALO algorithm is proposed to tackle the above-mentioned problems. The SAALO algorithm first employs a roulette algorithm to optimize the random wandering function of ants. The original random wandering function of ants results in a large disparity in the adaptation values of each individual in the algorithm, necessitating numerous iterative calculations to find the optimal value. In the roulette algorithm, the probability of an individual being selected is directly proportional to its fitness. By calculating the fitness value of an individual to influence the migration direction of the population in the next step, the accuracy of the algorithm in finding the optimal solution is ensured, and the situation where some ants fall into the local minimum is also avoided. Finally, the simulated annealing algorithm is utilized for the migration judgment of the ant lion population. This enhances the population’s ability to escape from the local optimal solution and guarantees the reliability of the final solution results. The specific improvements are as follows.

#### 3.2.1. Roulette Algorithm

The roulette algorithm, also known as the proportional selection method [31], has the basic idea that the probability of each individual being selected is proportional to its fitness. The specific operations are as follows:

Step 1: The fitness value $f(x_{i}),i=1,2,\cdots ,m$ is calculated for each individual in the ant colony and m is the colony size.

Step 2: Calculate the probability of each individual being inherited into the next-generation population. For individuals with low fitness, take the inverse of their selection probability.

(33) $$P(x_{i})=\frac{\sum_{j=1}^{m}f(x_{j})}{f(x_{i})}$$

Step 3: Perform cumulative summation to calculate the cumulative probability of each individual.

(34) $$q_{i}=\sum_{j=1}^{i}P(x_{i}),q_{i}\in \left[0,1\right]$$

Step 4: A random value is taken to generate a uniformly distributed random number r in the interval [0, 1], defining the selection threshold $p_{i}$.

(35) $$p_{i}=r\times q_{i}$$

Step 5: If $p(x_{i})\geq p_{i}$, then the selection of an individual falls just inside the selection interval and individual $x_{i}$ is selected; otherwise, individual $x_{i}$ is selected such that $p_{\left(x_{k-1}\right)}<p_{k}\leq p_{\left(x_{k}\right)}$ holds.

Step 6: Repeat steps 4 and 5 m times.

Step 7: The random wandering function of the ants in the simulated annealing ant lion algorithm is shown in Equation (36).

(36) $$rw(t)=\sum_{i=0}^{t}\left(rw_{i}+x_{i}\right)$$

#### 3.2.2. Simulated Annealing Algorithm

The simulated annealing algorithm is modeled on the solid temperature drop process, and the Metropolis criterion is used to determine whether to accept a new result [19]. The algorithm can quickly search for a globally optimal solution or it can be mutated during the search process, which significantly improves the algorithm’s ability to escape from local optimal solutions. The description of the Metropolis criterion is as follows:

First, calculate the difference between the temperature at that moment and the previous moment, that is, the difference in the fitness function, as shown in Equation (37):

(37) $$\Delta tem=f\left(x_{t+1}\right)-f\left(x_{t}\right)$$

If Δtem<0, the solution at the moment t+1, is accepted as the new solution; otherwise, the probability of whether the solution is chosen as the new solution is calculated, and the probability formula is shown in the following equation:

(38) $$p=\left\{\begin{array}{cc} 1 & ,E_{Tnew}<E_{Tcur}\\ \exp(-\frac{E_{Tnew}-E_{Tcur}}{T}) & ,E_{Tnew}\geq E_{Tcur} \end{array}\right.$$

In Equation (38), p denotes the probability of accepting the solution at the moment t+1 as a new solution; $E_{Tnew}$ denotes the state energy at the current temperature tem. $E_{Tcur}$ denotes the state energy at the previous temperature tem-1.

In the SAALO algorithm, a simulated annealing algorithm is used to determine whether the ant lion population falls into local optimum by calculating the difference of the fitness function corresponding to the ΔT value in the simulated annealing algorithm, and at the same time with the help of the simulated annealing algorithm’s ability to jump out of the local optimum to enhance the ability of the global optimality search of the SAALO algorithm.

#### 3.2.3. SAALO Algorithm Flow

The process of using the SAALO algorithm to solve integer ambiguities is as follows:

Step 1: System initialization. Initialize the number of individuals, individual dimensions, maximum number of iterations, and search space of the ant and ant lion populations.

Step 2: Population initialization. Determine the search space range and initial position of the ant population based on the ambiguity floating-point solution. Then, calculate the initial adaptation value of the ants and the size of the traps, which will be used to determine the position of the ants after random wandering in the next step.

Step 3: Calculate the positions of the ant population. The ant population randomly wanders to search for the optimal solution using the random wandering function improved by the roulette algorithm. Additionally, the ants will also wander to a position in the search space determined by the ant lion trap of a larger size.

Step 4: Calculate the fitness value of the ant colony at the current moment. Then, provide the locations searched by all the ants at the current moment to the ant lion swarm.

Step 5: Determine the locations of the ant and ant lion populations at the next moment. The ant lion preys on the ants and moves to the current locations of the ants. Compare the current locations of the ant lion population with those at the previous moment and use the simulated annealing algorithm to determine whether the random wandering is valid. If the wandering is valid, adopt the post-wandering locations as the new locations of the ant lion population; if the wandering is invalid, continue to use the pre-wandering locations of the ant lion population. Then, calculate the size of the ant lion trap, which is used to predict the range of the next random wandering of the ants.

Step 6: Check whether the loop has reached the maximum number of iterations. If not, return to Step 3; if so, end the loop and output the optimal solution.

**Figure 2:**
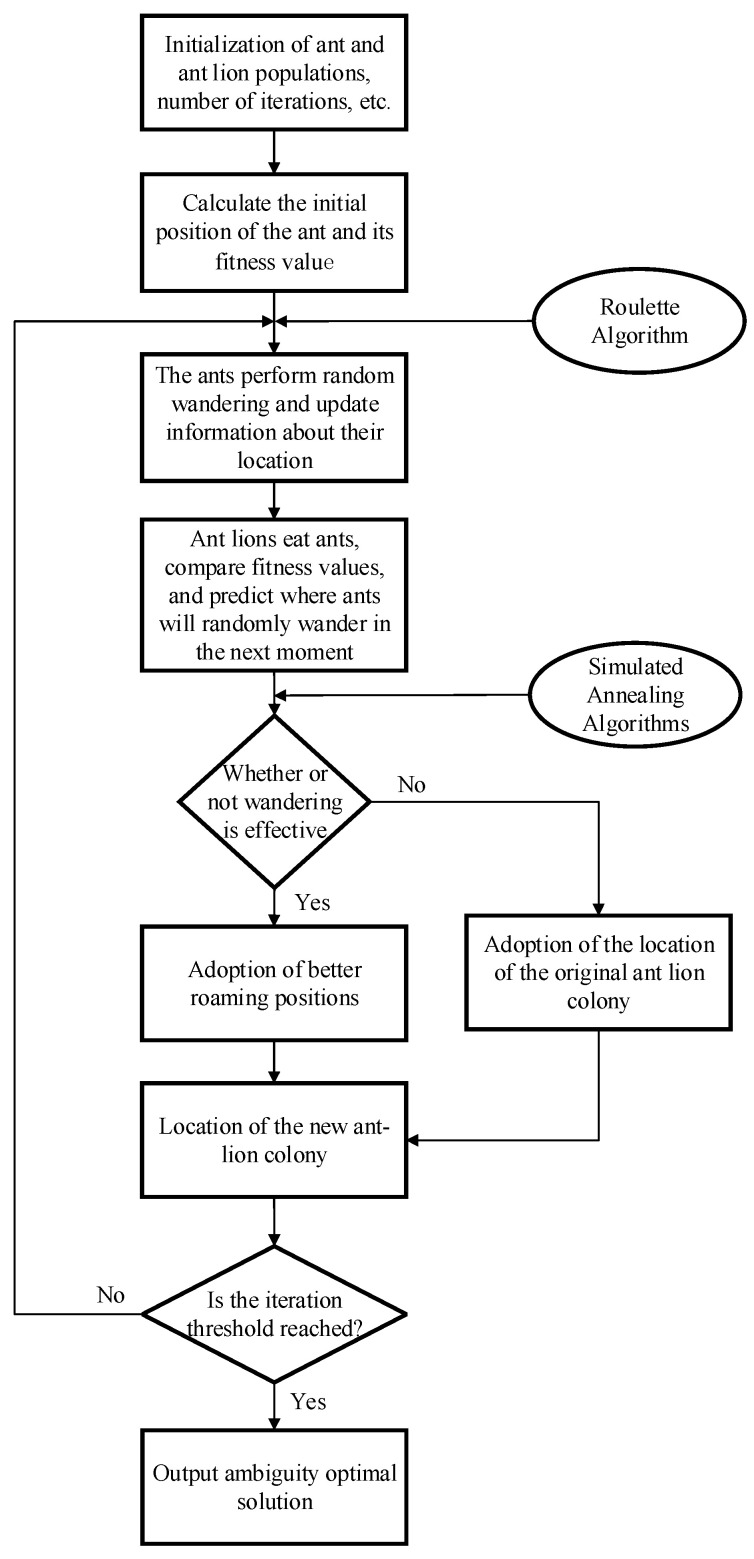
Flowchart of the SAALO algorithm.

The SAALO algorithm flowchart is presented in Figure 2.

### 3.3. SAALO Algorithm Performance Experiment and Result Analysis

#### 3.3.1. Test Functions and Evaluation Criteria

In this paper, twelve internationally recognized benchmark test functions are employed for testing, as presented in Table 1. F1–F5 are five single-peak test functions with singularities. These functions can be used to evaluate the algorithm’s convergence and explore its local search ability. F6–F12 are seven multi-peak test functions with multiple optima. Among these optimal solutions, one is globally optimal and the others are locally optimal. These multi-peak functions can be utilized to assess the algorithm’s global search capability [32]. The simulation experiment is conducted on a Windows 10 operating system. Given the random nature of the optimization algorithm, each experiment is independently repeated 30 times. The maximum number of iterations is set to 500, and the population size is 30. To intuitively evaluate the performance of the optimization algorithm, the mean and standard deviation are calculated based on the data from the 30 experiments. The mean can reflect the algorithm’s ability to find the optimal solution, while the standard deviation can indicate the algorithm’s stability during the optimization process.

#### 3.3.2. Comparison with Different Optimization Algorithms

**Figure 3:**
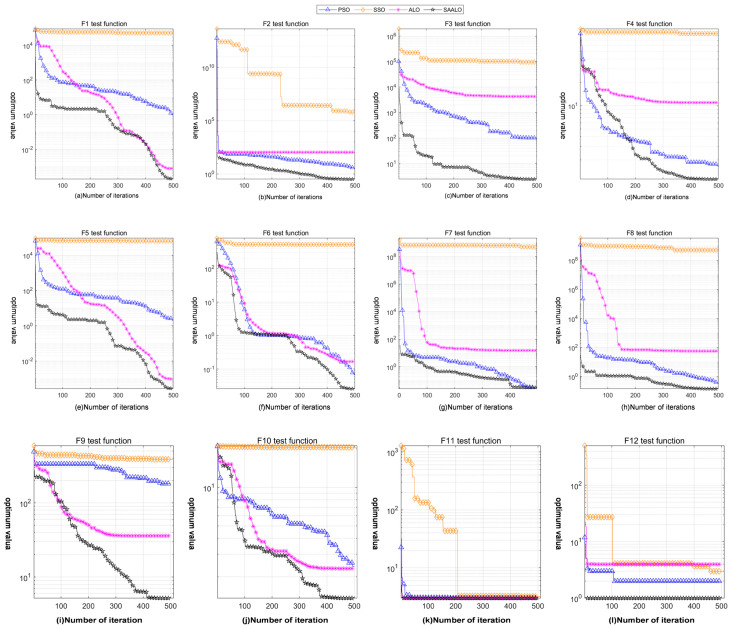
Iteration effect comparison among different optimizer algorithms on F1 to F12 test functions.

To verify the optimization ability of the SAALO optimization algorithm, this paper compared it with the Particle Swarm Optimization (PSO) algorithm, the Spherical Search Optimizer (SSO) algorithm, and the Ant Lion Optimizer (ALO) algorithm. Thirty independent experimental iterations are carried out to calculate the mean and standard deviation. The results are presented in Table 2. Additionally, a set of iterative data were randomly selected for graphical representation, as presented in Figure 3.

Algorithm parameter settings: the maximum number of iterations for each algorithm is 500, and the number of populations is 30; the individual learning factor $c_{1}$ in PSO is 2, the population learning factor $c_{2}$ is 2, and the particle velocity $V_{\max}$ is 6; the initial fitness counter FES in SSO is 0; the number of uniformly randomized distributions rw in the random wandering function r in ALO and SAALO takes the value in the range of [0, 1].

In the implementation of the SAALO algorithm, the initial temperature $T_{0}$ is set to 100, a parameter that critically influences the search efficiency. A value that is too low for $T_{0}$ can lead to premature convergence of the algorithm, whereas an excessively high value increases the computational time. The cooling rate, alpha, is established at 0.99, playing a pivotal role in determining the exploration capacity of the algorithm throughout the search process. The termination temperature $T_{\min}$ is designated as 1 × 10^−8^. A higher $T_{\min}$ necessitates a greater number of iterations, thereby extending the time consumption, whereas a lower $T_{\min}$ accelerates convergence but may compromise the algorithm’s global search capability. By meticulously configuring these three parameters, the SAALO algorithm can efficiently navigate the search space and achieve commendable performance in global optimization tasks.

From the standard deviation and mean values in Table 2, it is evident that in the single-peak test functions F1–F5, when compared with the PSO, SSO and ALO algorithms, the SAALO algorithm exhibits the optimal optimization ability and convergence speed. Moreover, its optimization performance in the single-peak test functions is more stable. In the multi-peak test functions F6–F12, the SAALO algorithm outperforms the PSO, SSO and ALO algorithms in terms of optimization ability and convergence speed. Among them, the results of the PSO and SAALO algorithms do not vary significantly when testing the F7 function, as presented in Figure 3g. However, the iteration speed of the SAALO algorithm is superior to that of the PSO algorithm. In addition, when testing the F11 function, there is not a significant difference in the test results of the PSO, ALO and SAALO algorithms, as presented in Figure 3k. However, the SAALO algorithm has a faster iteration speed than the PSO and ALO algorithms.

## 4. Numerical and Experimental Analysis

### 4.1. Three-Dimensional Numerical Analysis

To conduct simulation analysis experiments, we used a famous arithmetic example presented by Jonge [24,29,33]. We compared four algorithms in total: the ALO, the SAALO, the LAMBDA and the MLAMBDA, and analyzed their solution performance. The double-difference integer ambiguity floating-point resolution and covariance matrix in 3D are as follows:

$${\overset{⌢}{N}}_{3}=\left[\begin{array}{c} 5.45\\ 3.1\\ 2.97 \end{array}\right],Q_{\overset{⌢}{N}}=\left[\begin{array}{ccc} 6.29 & 5.978 & 0.544\\ 5.978 & 6.292 & 2.34\\ 0.544 & 2.34 & 6.288 \end{array}\right]$$

From matrix $Q_{\overset{⌢}{N}}$, it can be seen that the correlation between integer ambiguity is strong and needs to be down-correlated. After the down-correlation operation, the floating-point solution and the covariance matrix are transformed as follows:

$${\overset{⌢}{N}}_{Z}=\left[\begin{array}{c} 4.57\\ 10.02\\ 2.35 \end{array}\right],Q_{{\overset{⌢}{N}}_{Z}}=\left[\begin{array}{ccc} 4.476 & 0.334 & 0.230\\ 0.334 & 1.146 & 0.082\\ 0.230 & 0.082 & 0.626 \end{array}\right]$$

**Figure 4:**
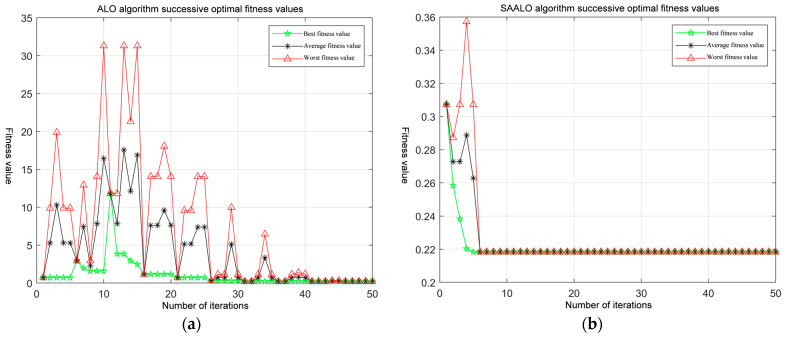
Comparison of convergence performance of different algorithms: (**a**) fitness evolution curve of ALO algorithm; (**b**) fitness evolution curve of SAALO algorithm.

Let the initial number of the ant population be 5, the population dimension be 3, and the maximum number of iterations be 50. Compare the ALO algorithm, the SAALO algorithm, the LAMBDA algorithm, and the MLAMBDA algorithm to analyze the search performance of the new algorithm. Among them, the adaptation change curves of the ALO algorithm and the SAALO algorithm are presented in Figure 4.

As can be seen from Figure 4, during the ambiguity search process, the adaptation values of the ALO algorithm and the SAALO algorithm decrease as the ant lion colony approaches the region of the optimal ambiguity. A number of experiments show that the ALO algorithm can reach the optimal fitness value region after about 41 iterations. After simulated annealing, the SAALO algorithm improves its ability to escape from the local optimal value. It will jump out of the region after the fitness value reaches the optimal value region and then conduct a new search to ensure the accuracy of the optimal solution. It can reach the optimal fitness value region in about six iterations near the optimal value.

**Figure 5:**
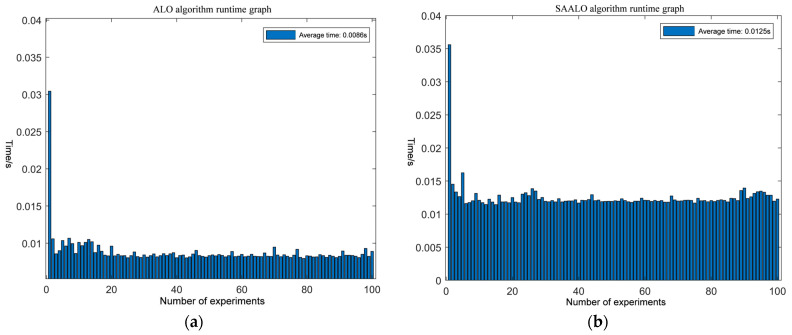
Comparison of solving time for different algorithms: (**a**) running time diagram of the ALO algorithm; (**b**) running time diagram of the SAALO algorithm.

To have a more accurate understanding of the solution time for each experiment, the computation times of the ALO and SAALO algorithms are presented in a bar chart in Figure 5.

As can be seen from Figure 5, by comparing the running times of the three algorithms in each trial, the ALO algorithm has the shortest solution time, which is concentrated around 0.0086 s each time. The SAALO algorithm has a slightly longer solution time, at approximately 0.0125 s each time.

To compare and analyze the accuracy and reliability of the algorithms in three dimensions, the above four algorithms are each run 100 times, with the maximum number of iterations set to 50 each time. The solution results, average solution times, and solution success rates are presented in Table 3.

As can be seen from Table 3, although the ALO algorithm is fast, the randomness in its solving process is too high, resulting in unstable solving results and a low solving success rate. For the SAALO algorithm, due to the introduction of the simulated annealing algorithm, even if it gets trapped in a local optimum during the search process, it can promptly escape from the local optimal region and then search for a better optimal solution for the ambiguity. When compared with the LAMBDA and the MLAMBDA, the SAALO slightly outperforms them in terms of solving time. It is 0.0496 s faster than the LAMBDA algorithm and 0.01 s faster than the MLAMBDA algorithm. In terms of the solving success rate, it is on par with the two algorithms.

Although the SAALO algorithm can effectively solve the three-dimensional ambiguity problem, with the application of multi-frequency and multi-system technologies, high-dimensional ambiguity resolution problems will become predominant in the actual RTK localization process. Therefore, high-dimensional ambiguity resolution experiments are conducted in Section 4.2.

### 4.2. Multi-Dimensional Numerical Analysis

With the development of satellite systems in different countries, high-precision positioning boards supporting multiple systems have become the mainstream. The introduction of multiple systems significantly increases the number of satellites involved in the resolution process and raises the complexity of ambiguity search and resolution. Therefore, the efficiency of multi-dimensional integer ambiguity determination is one of the crucial indices of an integer ambiguity determination algorithm. In this paper, 3D, 6D, and 12D ambiguity resolution experiments are carried out for the SAALO algorithm. Among them, the 3D experimental data iares consistent with that in Section 4.1, and the 12D and 6D floating-point solution matrices are as follows:

$$\begin{array}{ll} {\overset{⌢}{N}}_{6}= & [\begin{array}{ccc} 9.9984 & 30.9995 & -32.0003 \end{array}\\ & \begin{array}{ccc} -35.0022 & 9.9955 & -43.0015 \end{array}{]}^{T} \end{array}\;\begin{array}{ll} {\overset{⌢}{N}}_{12}= & [\begin{array}{ccc} -28491 & 65753 & 38830 \end{array}\\ & \begin{array}{lll} 5003.7 & -29196 & -297.6589 \end{array}\\ & \begin{array}{ccc} -22201 & 51236 & 30258 \end{array}\\ & \begin{array}{ccc} 3899.4 & -22749 & -159.2788 \end{array}{]}^{T} \end{array}$$

**Figure 6:**
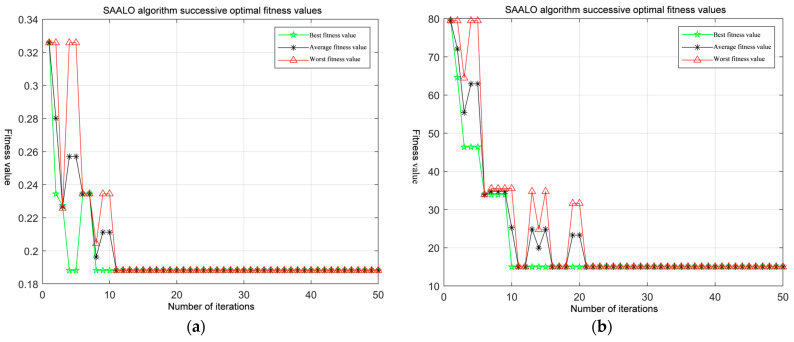
Comparison of high-dimensional convergence performance of different algorithms: (**a**) fitness evolution curve of 6D SAALO algorithm; (**b**) fitness evolution curve of 12D SAALO algorithm.

Let the initial number of the ant population be 5 and the maximum number of iterations be 50. The fitness change curve of the solution process is presented in Figure 6.

As presented in Figure 6a,b, regardless of whether it is for 6D or 12D integer ambiguity resolution, the SAALO algorithm can search for the optimal ambiguity solution again in the subsequent period, even if it falls into a local optimum briefly, thus escaping from the local optimum. The number of convergence generations of the SAALO algorithm gradually increases with the increase in the number of dimensions of the integer ambiguity. During the 6D ambiguity resolution, the number of convergence generations of the algorithm increases to 11. In the 12D resolution process, the algorithm converges at 21 generations.

**Figure 7:**
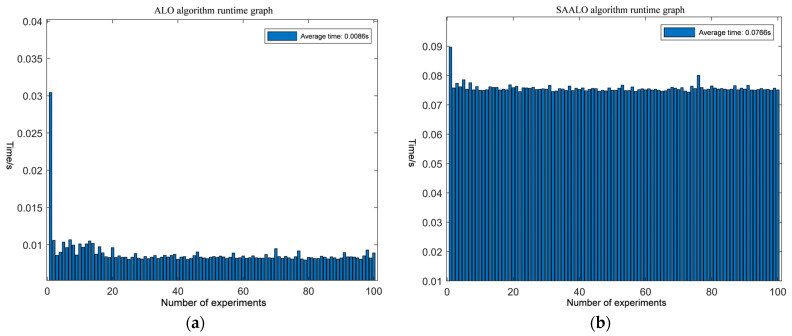
Comparison of high-dimensional solving times for different algorithms: (**a**) running time diagram of 6D SAALO algorithm; (**b**) running time diagram of 12D SAALO algorithm.

The running times of the SAALO algorithm for 6D and 12D ambiguity resolution are presented in Figure 7a,b. As the number of ambiguity dimensions increases, the complexity of ambiguity resolution rises, leading to a corresponding increase in the resolution time. For 6D ambiguity resolution, the single-run time of the SAALO algorithm is concentrated around 0.0325 s. In contrast, for 12D ambiguity resolution, the single-run time of the SAALO algorithm is concentrated around 0.0766 s.

To compare and analyze the accuracy and reliability of the SAALO algorithm in different dimensions, the SAALO algorithm was run 100 times for 3D, 6D, and 12D ambiguities, with the maximum number of iterations is 50. The average solution time and solution success rate are presented in Table 4.

As can be seen from Table 4, as the number of ambiguity dimensions increases, the average solution time of the algorithm rises from 0.0125 s in 3D to 0.0325 s in 6D, and further increases to 0.0766 s in 12D. Meanwhile, the success rate of ambiguity resolution drops from 100% in 3D to 99% in 6D, and then to 98% in 12D.

Although the average solution time of the SAALO algorithm increases and the solution success rate decreases with the increase in the number of ambiguity dimensions, the algorithm’s solution speed and success rate can still meet the engineering requirements.

### 4.3. Test Analysis

**Figure 8:**
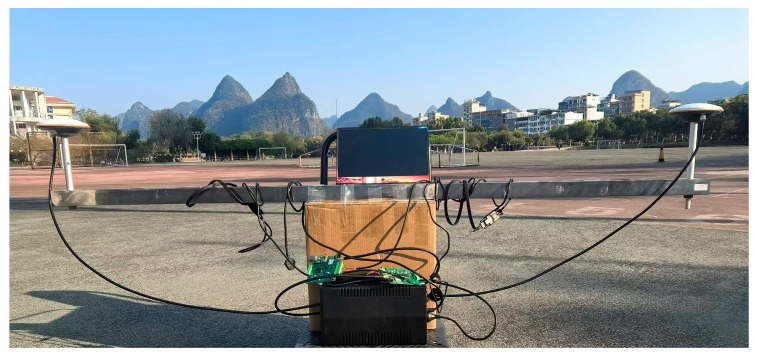
Data collection site diagram.

To test the performance of the algorithm in a practical application environment, the SAALO algorithm is applied to the RTK experiment. The experiment consists of two tests. In the first test, data are acquired using a single-frequency U-blox receiver, and 644 epochs of data are collected. The distance between the reference station and the mobile station is 2.1 m, and the data collection site is the track field of the Huajiang Campus, Guilin University of Electronic Science and Technology, Guilin, China. Figure 8 shows the schematic diagram of the data collection site.

**Figure 9:**
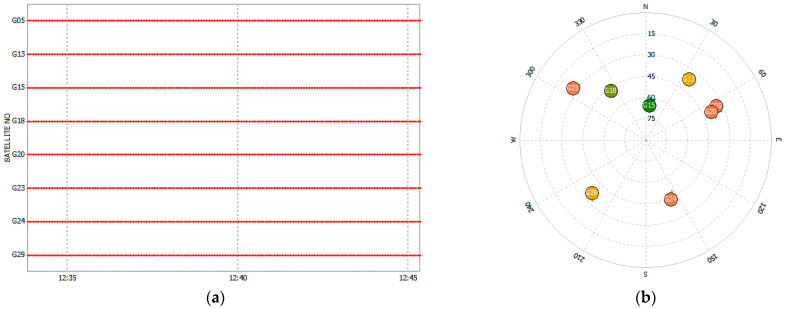
Observation data analysis: (**a**) visible satellite condition; (**b**) visible satellite sky map.

Through the analysis of the mobile-station data, a satellite elevation cutoff angle of 15° was adopted. The visibility of satellites at the mobile station is presented in Figure 9. In the fig., the horizontal axis represents time and the vertical axis represents the GPS satellite numbers.

In this experiment, only single-frequency GPS data were used for the calculation. As can be seen from Figure 9, a total of eight GPS satellites were visible during the data collection period, and all these eight visible satellites were involved in the subsequent calculation process.

**Figure 10:**
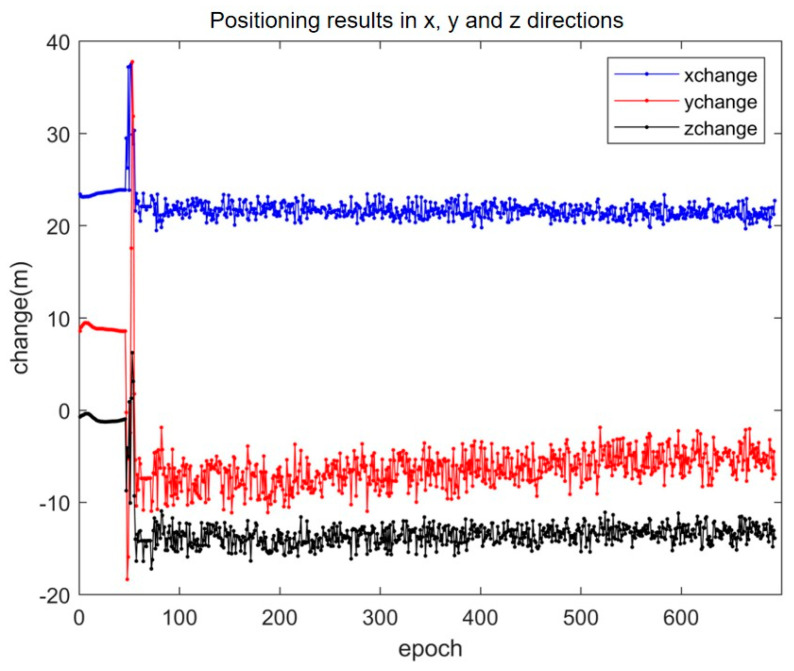
The positioning effect in x, y and z directions when the ambiguity of the integer is not fixed.

The resolution of integer ambiguity is crucial for satellite positioning accuracy. As presented in Figure 10, the data collected from 644 epochs were used for ambiguity solving in the RTK localization experiment. Without resolving the integer ambiguity, the positioning effect is very poor and fails to meet the engineering requirements.

Subsequently, the SAALO algorithm is used to resolve the integer ambiguity. As Presented in Table 5, the SAALO algorithm processes the solution results of 644 epochs of single-frequency GPS data. It can be seen that the SAALO algorithm shows better performance in resolving integer ambiguity. It has a shorter solving time compared to the LAMBDA algorithm, and its solving success rate is on average 5.9% higher than that of the LAMBDA algorithm.

**Figure 11:**
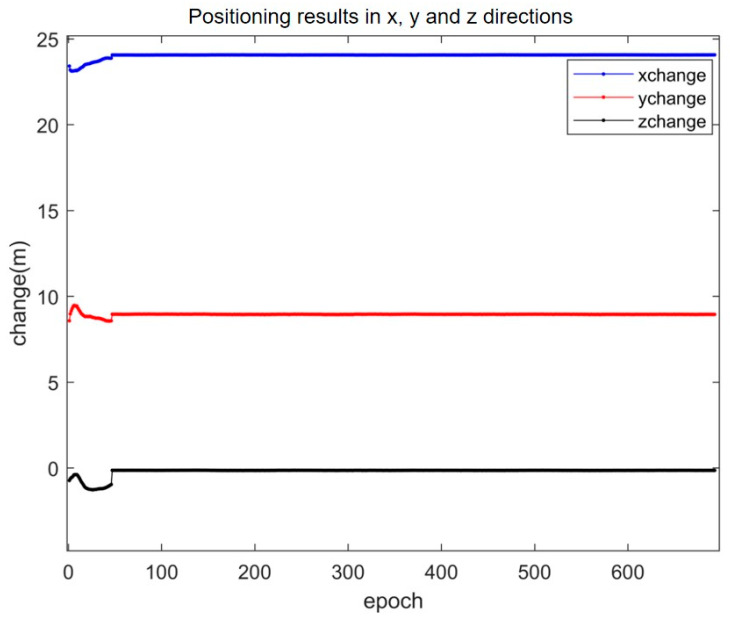
The positioning effect in x, y and z directions when the ambiguity of the integer is fixed.

As can be seen from Figure 11, the SAALO algorithm successfully converges within the first 50 epochs. This enables it to effectively resolve the integer ambiguity problem and enhance the positioning accuracy in the x, y and z directions.

**Figure 12:**
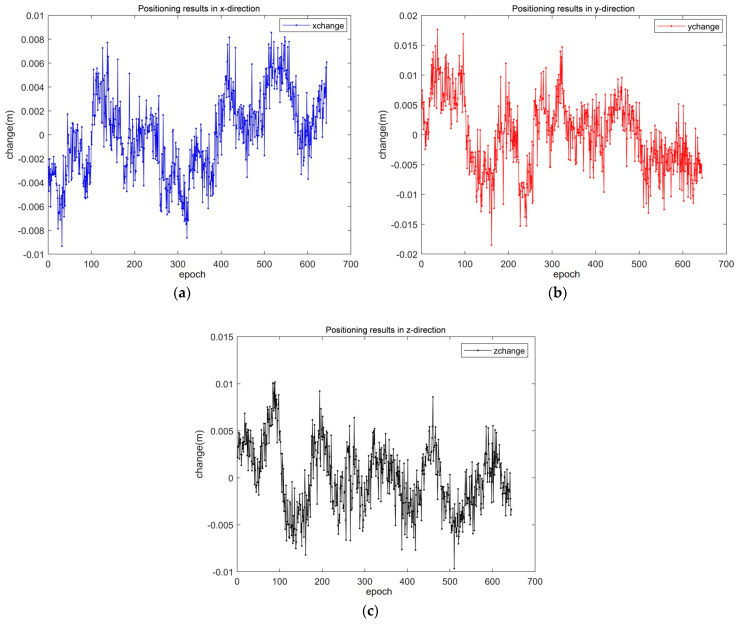
Positioning results: (**a**) positioning results in x direction; (**b**) positioning results in y direction; (**c**) positioning results in z direction.

The details of its positioning results are presented in Figure 12. As can be seen from Figure 12, the errors of the SAALO algorithm in the x, y and z directions are ±0.8 cm, ±1.5 cm and ±1 cm, respectively, which allows for centimeter-level positioning.

In the second experiment, the hkws and hksl base stations in Hong Kong, China, are utilized. The hksl base station is the reference station. The hkws base station is the rover station. The baseline length is 42.7 km. Table 6 presents the relevant information of the two base stations used in the experiment.

The data files of the same day from the two base stations are downloaded and parsed using the RTKLIB (Real-Time Kinematic Library) program. Table 7 shows the relevant specifications of the RTKLIB_2.4.3 software used in the experiment.

The traditional LAMBDA algorithm and the SAALO algorithm are used for integer ambiguity resolution; 1000 epochs of data are selected for resolution, and the success rates and resolution times of the two algorithms are compared, as presented in Table 8.

From Table 5 and Table 8, it can be observed that the success rate of resolving the long-baseline integer ambiguity is much lower than that of the short-baseline case. This is because the increase in baseline length expands the range of ambiguity, resulting in a decrease in the resolution success rate. However, the SAALO algorithm proposed in this paper still demonstrates better performance in resolving integer ambiguity. The SAALO algorithm has a shorter solution time compared to the LAMBDA algorithm, and its solution success rate is 5.2% higher than that of the LAMBDA algorithm.

For single-frequency and single-system scenarios, the SAALO algorithm has a sufficiently fast solving speed to ensure real-time RTK resolution effectively. The high success rate of resolution and positioning accuracy guarantee the precision of RTK positioning. This algorithm can meet the requirements of short- and medium-baseline single-frequency and single-system engineering resolution, and it holds significant advantages in both theoretical research and practical applications.

## 5. Conclusions and Future Works

Through conducting ambiguity resolution experiments using the SAALO algorithm in different dimensions and applying the algorithm to the engineering resolution of single-frequency and single-system scenarios, the following conclusions are drawn based on an analysis of the SAALO algorithm in terms of its running time, resolution success rate, and positioning accuracy.

(a) In the case of three-dimensional ambiguity resolution, the SAALO algorithm can outperform the LAMBDA algorithm and the MLAMBDA algorithm in terms of average solution time while ensuring the resolution success rate. It is 0.0496 s faster than the LAMBDA algorithm and 0.01 s faster than the MLAMBDA algorithm.

(b) In the case of high-dimensional ambiguity resolution, as the ambiguity dimension increases, the difficulty of ambiguity resolution rises, and the success rate of the SAALO algorithm decreases slightly. However, the resolution success rate can still be maintained at 98% and above. Additionally, although the computation time of the SAALO algorithm becomes longer as the ambiguity dimension increases, the resolution time still meets the real-time requirements.

(c) In single-frequency and single-GPS system engineering, the SAALO algorithm is applied to resolve the integer ambiguity under short- and medium-baseline conditions. When the baseline length is 42.7 km, the average solution time of the SAALO algorithm is slightly shorter than that of the LAMBDA algorithm, and its solution success rate is 5.2% higher than that of the LAMBDA algorithm. The results indicate that the SAALO algorithm exhibits good resolution ability in the engineering applications of short and medium baselines.

In the subsequent research work, the SAALO algorithm will be further optimized to address the issue of the reduced success rate of ambiguity resolution for medium- and long-baselines. Meanwhile, the SAALO algorithm will also be applied to multi-frequency and multi-system engineering solutions.

## Figures and Tables

**Table 1:** Test functions.

Function Expression (Math.)	Dimension (Math.)	Search Scope	Optimal Solution
$F_{1}(x)=\sum_{i=1}^{n}x_{i}^{2}$	30	[−100, 100]	0
$F_{2}(x)=\sum_{i=1}^{n}\left|x_{i}\right|+\prod_{i=1}^{n}\left|x_{i}\right|$	30	[−10, 10]	0
$F_{3}(x)=\sum_{i=1}^{n}{\left(\sum_{j-1}^{i}x_{j}\right)}^{2}$	30	[−100, 100]	0
$F_{4}(x)=\underset{i}{\max}\left\{\left|x_{i}1\leq i\leq n\right|\right\}$	30	[−100, 100]	0
$F_{5}(x)=\sum_{i=1}^{n}{\left(\left[x_{i}+0.5\right]\right)}^{2}$	30	[−100, 100]	0
$F_{6}(x)=\frac{1}{4000}\sum_{i=1}^{n}x_{i}^{2}-\prod_{i=1}^{n}\cos(\frac{x_{i}}{\sqrt{i}})+1$	30	[−600, 600]	0
$\begin{array}{l} F_{7}(x)=\frac{\pi }{n}\left\{10\sin(\pi y_{1})+\sum_{i=1}^{n-1}{\left(y_{i}-1\right)}^{2}\left[1+10\sin^{2}(\pi y_{i+1})\right]+{\left(y_{n}-1\right)}^{2}\right\}\\ +\sum_{i=1}^{n}u(x_{i},10,100,4)\\ y_{i}=1+\frac{x_{i}+1}{4}\\ u(x_{i},a,k,m)=\left\{\begin{array}{cc} k{\left(x_{i}-a\right)}^{m} & ,x_{i}>a\\ 0 & ,-a<x_{i}<a\\ k{\left(-x_{i}-a\right)}^{m} & ,x_{i}<-a \end{array}\right. \end{array}$	30	[−50, 50]	0
$\begin{array}{l} F_{8}(x)=0.1\left\{\sin^{2}(3\pi x_{1})+\sum_{i=1}^{n}{\left(x_{i}-1\right)}^{2}\left[1+\sin^{2}(3\pi x_{i}+1)\right]+{\left(x_{n}-1\right)}^{2}\left[1+\sin^{2}(2\pi x_{n})\right]\right\}\\ +\sum_{i=1}^{n}u(x_{i},5,100,4) \end{array}$	30	[−50, 50]	0
$F_{9}(x)=\sum_{i=1}^{n}\left[x_{i}^{2}-10\cos(2\pi x_{i})+10\right]$	30	[−5.12, 5.12]	0
$F_{10}(x)=-20\exp(-0.2\sqrt{\frac{1}{n}\sum_{i=1}^{n}x_{i}^{2}})-\exp\left(\frac{1}{n}\sum_{i=1}^{n}\cos(2\pi x_{i})\right)+20+e$	30	[−32, 32]	0
$\begin{array}{ll} F_{11}(x) & =\left[1+{\left(x_{1}+x_{2}+1\right)}^{2}\left(19-14x_{1}+3x_{1}^{2}-14x_{2}+6x_{1}x_{2}+3x_{2}^{2}\right)\right]\\ & \times \left[30+{\left(2x_{1}-3x_{2}\right)}^{2}\times (18-32x_{1}+12x_{1}^{2}+48x_{2}-36x_{1}x_{2}+27x_{2}^{2})\right] \end{array}$	2	[−2, 2]	3
$F_{12}(x)={\left(\frac{1}{500}+\sum_{j=1}^{25}\frac{1}{j+\sum_{i=1}^{2}{\left(x_{i}-a_{ij}\right)}^{6}}\right)}^{-1}$	2	[−65, 65]	1

**Table 2:** Comparison of performance test results of four algorithms.

Function (Math.)	PSO		SSO		ALO		SAALO	
	Average Value	Standard Deviation	Average Value	Standard Deviation	Average Value	Standard Deviation	Average Value	Standard Deviation
$F_{1}$	2.607	1.612	5.485 × 10^4^	5.063 × 10^6^	1.445 × 10^−3^	1.035 × 10^−3^	4.73 × 10^−4^	2.316 × 10^−4^
$F_{2}$	4.900	1.452	1.173 × 10^9^	2.008 × 10^9^	54.403	49.52	1.324	1.201
$F_{3}$	1.905 × 10^2^	65.36	7.338 × 10^4^	1.405 × 10^4^	4.349 × 10^3^	2.233 × 10^3^	1.698	0.573
$F_{4}$	1.960	0.299	83.707	3.884	16.533	3.787	1.025	0.280
$F_{5}$	2.253	1.097	5.181 × 10^4^	5.982 × 10^3^	1.34 × 10^−3^	1.825 × 10^−3^	4.849 × 10^−4^	2.601 × 10^−4^
$F_{6}$	0.123	0.038	4.59 × 10^2^	39.740	0.060	0.031	0.036	0.024
$F_{7}$	0.066	0.065	3.422 × 10^8^	1.093 × 10^8^	11.205	3.659	0.066	0.061
$F_{8}$	0.516	0.223	7.012 × 10^8^	1.922 × 10^8^	29.195	21.696	0.0620	0.066
$F_{9}$	165.85	33.705	382.26	24.383	78.337	23.648	5.265	1.103
$F_{10}$	2.736	0.404	20.308	0.238	5.037	3.254	1.531	0.201
$F_{11}$	3.000	6.122 × 10^−15^	3.554	0.834	3.000	6.242 × 10^−13^	3.000	6.762 × 10^−13^
$F_{12}$	2.966	2.859	2.952	1.721	2.187	1.407	0.998	3.997 × 10^−16^

**Table 3:** Comparison results of three-dimensional solving time for five algorithms.

Experimental Algorithms	Norm		
	Solution Result	Average Solving Time/s	Solution Success Rate/%
ALO	5, 3, 4	0.0086	56
SAALO	5, 3, 4	0.0125	100
LAMBDA	5, 3, 4	0.0621	100
M-LAMBDA	5, 3, 4	0.0225	100

**Table 4:** Comparison of the solution time of the SAALO algorithm in different dimensions.

Example Algorithm	Dimensionality	Norm		
		Solution Result	Average Solving Time/s	Solution Success Rate/%
SAALO	3	5, 3, 4	0.0125	100
	6	−28,506, 65,833, 38,880, 5008, −29,210, −257	0.0325	99
	12	28,451, 65,749, 38,814, 5025, −29,165, −278, −22,170, 51,233, 30,245, 3916, −22,725, −144	0.0766	98

**Table 5:** Results of the SAALO algorithm.

Example Algorithm	Norm				
	Total Number of Epoch/Each	The Number of Successful Epoch Is Solved/Each	Solution Success Rate/%	Solve the Total Time/s	Calculate the Average Time/s
SAALO	644	642	99.7	8.587	0.0133
LAMBDA	644	604	93.8	9.325	0.0145

**Table 6:** Hkws and hksl base station parameter table.

Station	Cartesian Coordinate			Receiver Type	Antenna Type
	X	Y	Z		
hkws	−2,430,579.0108	5,374,285.6738	2,418,956.3331	Leica GR50	Leica AR25.R4 + LEIT
hksl	−2,393,382.4157	5,393,861.1745	2,412,592.4105	Leica GR50	Leica AR25.R4 + LEIT

**Table 7:** RTKLIB software parameter table.

Software Name	Version	Developer	Supports GNSS System	Positioning Mode	File Format	Communication Protocol
RTKLIB	2.4.3	Tomoji Takasu	GPS, GLONASS, Galileo, BeiDou, etc.	RTK, PPP, Static, dynamic	RINEX, RTCM, NMEA, etc.	NTRIP, TCP/IP, Serial port, etc.

**Table 8:** Results of the SAALO and LAMBDA algorithms.

Example Algorithm	Norm				
	Total Number of Epoch/Each	The Number of Successful Epoch Is Solved/Each	Solution Success Rate/%	Solve the Total Time/s	Calculate the Average Time/s
LAMBDA	1000	524	52.4	14.968	0.0150
SAALO	1000	576	57.6	14.271	0.0143

## Data Availability

The raw data supporting the conclusions of this article will be made available by the authors upon request.

## Abbreviations

The following abbreviations are used in this manuscript:

ALO	Ant Lion Optimizer
SAALO	Simulated Annealing Ant Lion Optimizer
LAMBDA	Least-square Ambiguity Decorrelation Adjustment
MLAMBDA	Modified Least-square Ambiguity Decorrelation Adjustment
ICSO	Improved Chicken Swarm Optimization
GNPS	Global Navigation and Positioning Systems
RTK	Real-Time Kinematic
GA	Genetic Algorithm
ILS	Integer Least Squares
IA	Integer Aperture
OIA	Optimal Integer Aperture
PSO	Particle Swarm Optimization
DGPS	Differential Global Position System
ACO	Ant Colony Optimization
AWDE	Adaptive Weighting Differential Evolution
EKF	Extended Kalman Filter
LS	Least Squares
DIAFV	Double-Difference Integer Ambiguity Floating-Point Values
SSO	Spherical Search Optimizer
RTKLIB	Real-Time Kinematic Library
